# Frequency of ABO blood groups and RhD factor in the female population of District Peshawar

**DOI:** 10.12669/pjms.314.6640

**Published:** 2015

**Authors:** Rubina Nazli, Jamila Haider, Mohammad Akmal Khan, Tasleem Akhtar, Hina Aslam

**Affiliations:** 1 Dr. Rubina Nazli, MBBS, PhD. Associate Professor of Biochemistry, Institute of Basic Medical Sciences, Khyber Medical University, Peshawar, Pakistan; 2 Jamila Haider, BS (Microbiology), PhD Scholor. Centre of Biotechnology & Microbiology, University of Peshawar, Peshawar, Pakistan; 3 Dr. Mohammad Akmal Khan, MBBS, MCPS, FCPSI. Psychiatry Unit, Hayatabad Medical Complex, Peshawar, Pakistan; 4 Dr. Tasleem Akhtar, M. Phil, PhD. Associate Professor, Chemistry Department, Sheringal University, Dir Upper, Khyber Pakhtunkhwa, Pakistan; 5 Dr. Hina Aslam, MBBS, FCPS-1. Gynea Unit, Hayatabad Medical Complex, Peshawar, Pakistan

**Keywords:** ABO blood group, Rh factor, Female population

## Abstract

**Objective:**

To determine the frequency of ABO blood group and Rhesus (Rh) D antigen in the females of “District” Peshawar, Khyber Pakhtunkhwa Province, Pakistan.

**Methods:**

This cross-sectional study was conducted on 429 women having pregnancy induced hypertension, admitted in the three teaching hospitals of Peshawar, over a period of one year. Blood sample was collected from each subject after taking informed consent. The antigen antibody agglutination slide test for “blood grouping (ABO)” and RhD factors was done by using IgM and IgG monoclonal reagents. The antisera used were from Biolaboratory, USA. Data was analyzed for percentage calculation.

**Results:**

The blood group distribution was 134 (31.2%), 43 (10.1%), 116 (27%), 136 (31.7%) for blood groups A, AB, O and B, respectively. Subjects having blood group B was slightly more dominant, followed by A and O, while blood group AB was rare in these females. Blood group A Rh negative is more in female 12 (37.5%) followed by group O 10 (31.3%), group B 09 (28.1%) and group AB 01 (3.1%).

**Conclusion:**

Frequency of “Rh-positive blood group” is B, A, O and AB, whereas the frequency of the most common Rh-negative blood group are A, O, B and AB respectively. The determination of the frequency of blood groups in the region would not only help in blood transfusion services, but also reduce the risk of erythroblastosis foetalis in the neonates.

## INTRODUCTION

Blood group is genetically predisposed. Until now 400 blood groups are reported but the most important in them are ABO and Rh.^1^ In transfusion, the most important blood groups are also ABO and Rh blood group system.^2^

ABO is the human blood group and it depends on presence of A or B genes.^3^ “ABO blood-group antigens are oligosaccharides attached to cell-surface glycoconjugates expressed by epithelia, endothelia and erythrocytes (RBCs) in primates”.^4^

The distribution of ABO and Rh blood groups vary from one race to another race; across the world in the population and within human subpopulations. Differences are present even in Pakistan due to racial differences.^5^ Blood group prevalence plays a role in evolution, genetics research, blood transfusion and organ transplantation. Modern medicine is also working on relationship of blood group with environment.^6^

The aim of the present study was to find out the frequency of different blood groups in the female population of District Peshawar, Khyber Pakhtunkhwa province Pakistan. Another purpose was to generate data for multipurpose future utilities.

## METHODS

A total of 429 females, were screened for blood grouping during a study on pregnancy induced hypertensive women after taking informed consent. Blood samples were taken by aseptic techniques and blood transfer to ethylene diamine tetra acetate (EDTA) containing tubes. The antigen antibody agglutination test was done for determination of blood grouping (ABO) and RhD factor using slide method. Biolaboratory, USA antisera were used in this study. The ABO monoclonal reagents are of hybridized immunoglobulins secreting mouse cell-line. IgM + IgG monoclonal reagents were used for determining RhD factor.

## RESULTS

Out of total 429 subjects, RhD positive blood groups were found in 397 (92.5%) and 32 (7.5%) were found to be RhD negative. ABO blood grouping in the subjects is shown in Table-I. Blood group distribution in the total sample was 134 (31.2%), 136 (31.7%), 116 (27%) and 43 (10.1%) for blood groups A, B, O and AB, respectively. The dominant blood group found in our study is blood group B followed by A and O, while blood group AB was rare in these females. Blood group A is common in RhD negative females 12(37.5%) and then group O 10(31.3%), group B 09(28.1%) and group AB 01(3.1%).

**Table-I:** ABO blood grouping pattern and RhD factor in females of district Peshawar.

Blood group	Total subjects N (%)	Rh + N (%)	Rh – N (%)
A	134(31.2)	122(30.7)	12(37.5)
B	136(31.7)	127(32.0)	09(28.1)
O	116(27.0)	106(26.7)	10(31.3)
AB	43(10.1)	42(10.6)	01(3.1)
Total	429(100)	397(100)	32(100)

## DISCUSSION

The frequency of ABO and RhD blood group vary from one population to another all over the world. Blood grouping is used in blood transfusion, because it is preferable for patients to receive blood of the same ABO and RhD group. It is also important in determining migration of races and in hereditary diseases.^5^ Some diseases are more common to develop in certain blood groups; hence relationship of different blood groups with diseases is important.^7^

Studies in different areas of Pakistan show the incidence and division of ABO and RhD blood groups in different regions.^8^,^9^ In our study, group B was more prevalent 136(31.7%), followed by group A 134(31.2%), group O 116(27%) and group AB 43(10.1%). While 92.5% blood groups were RhD positive and 7.5% were RhD negative.

In Pakistan, studies show variations in blood groups in different provinces. A study conducted in Sindh^10^ showed that blood group O was most prevalent as 36%. Group B (30%), group A (25%) and blood group AB as 9%. A study from “Baluchistan” reported^11^ blood group AB (7.57%) groups A (21.12%) group B (34.32%), and O as (37.07%).

A study from Rawalpindi and Islamabad^12^ had shown the percentages of various groups among female subjects, as 32.87% for blood group B, 31.91% for blood group O, 24.02% for blood group A, and 11.20% for blood group AB. Distribution of Rh positive and negative in the considered population was 92.45% and 7.55% respectively.

Blood group B Rh positive female subjects was found to be dominant (28.06%) followed by O (25.5%), A (24.50%) and group AB (9.43%) as reported in a study from Swat,^13^ Khyber Pakhtunkhwa Province (KPK).

Another study from Bannu KPK^14^ revealed that the distribution of ABO groups is in the order of 36.23% (B), 31.03% (A), 25.07% (O) and 7.67% (AB). The RhD positive and negative distribution in the studied population was 89.23% and 10.77% respectively.

Punjab and Khyber Pakhtunkhwa Province studies show blood group B as commonest. Our study shows similar results.^6^-^8^,^15^ In Sind and Baluchistan blood group O is found to be dominant which is contrary to our study.^10^,^11^,^16^

Studies conducted in different regions show that blood group O is more prevalent (46%) in USA^17^, Saudi Arabia^18^ (52%) and in the population of Iran^19^ (41.16%). The most prevalent group in Africans is B group while in Australians is O and A is the much commoner.^20^

In our study 92.5% blood groups were RhD positive and 7.5% were RhD negative. Rh positive male donors are 93% in Saudi Arabia.^18^ In the USA, 85% of the population are the Rh positive^17^, while in the British population^21^ 95% are Rh positive.

The result of our study matches with other studies done in different regions of Pakistan. Blood group RhD-Positive blood group is predominant group and its frequency is almost same.
